# Leptin Distribution in Rat Foetal and Extraembryonic Tissues in Late Gestation: A Physiological View of Amniotic Fluid Leptin

**DOI:** 10.3390/nu12092542

**Published:** 2020-08-21

**Authors:** Zhi Xin Yau-Qiu, Catalina Picó, Ana María Rodríguez, Andreu Palou

**Affiliations:** 1 Laboratory of Molecular Biology, Nutrition and Biotechnology (Group of Nutrigenomics and Obesity), University of the Balearic Islands (UIB), Palma de Mallorca, 07122 Balearic Islands, Spain; zx.yau@uib.es (Z.X.Y.-Q.); cati.pico@uib.es (C.P.); andreu.palou@uib.es (A.P.); 2 Health Research Institute of the Balearic Islands (IdISBa), Palma de Mallorca, 07010 Balearic Islands, Spain; 3 CIBER de Fisiopatología de la Obesidad y Nutrición (CIBERobn), Palma de Mallorca, 07122 Balearic Islands, Spain

**Keywords:** leptin, metabolic programming, amniotic fluid, gastric content, foetal stomach, placenta, late gestation

## Abstract

Prenatal leptin is key to regulating foetal growth and early metabolic programming. The presence of intact leptin in rat foetal (at late gestation) and neonatal (immediately after birth) stomach content and mucosa has been previously described, suggesting that it may act as a regulatory nutrient for the neonate rats, be internalised by the stomach, and play a physiological role early in life, which requires to be further investigated, including its origin. We aimed to study the ontogeny of the presence of leptin in the foetal stomach and key extraembryonic tissues in rats at late gestation (days 18–21). Leptin concentration was determined by enzyme-linked immunosorbent assay, and placental leptin immunolocalisation was analysed by immunohistochemistry. Leptin showed a sudden appearance in the amniotic fluid (AF) at day 20 of gestation, gastric content (swallowed AF), stomach, and umbilical cord, significantly increasing at day 21. Leptin levels in these fluids and tissues were positively correlated. In the placenta, leptin was detectable at all the studied days, but its localisation changed from widespread throughout the placenta at day 18 to well-defined in the labyrinth zone from day 19 onwards. The results support a possible internalisation of AF leptin by the immature stomach of near-term foetuses and suggest that changes in placental leptin localisation might help to explain the sudden appearance of leptin in AF at gestational day 20, with potential physiological significance regarding short-term feeding control and metabolic programming in the developing offspring.

## 1. Introduction

Gestation is a high-nutritional-demand process in which optimal macro- and micronutrient supply is pivotal for foetal development, as well as for extraembryonic tissue formation and maternal tissue expansion to support foetal growth [1]. Leptin has been described to play an important role in the regulation of many processes during pregnancy. Leptin from different sources, including the placenta and maternal circulation, participates in the modulation of the embryo implantation and innate and adaptive immunity, in the stimulation of nutrient uptake and protein synthesis to promote foetal growth, and in the mediation of the development of several foetal tissues [2,3,4]. It has been reported that deregulation of leptin production or availability during pregnancy could alter foetal growth through inappropriate placental development and function and program offspring to a higher risk of developing metabolic diseases, such as obesity [5,6,7]. The placenta is an important source of leptin, produced mainly by the syncytiotrophoblast cells of the labyrinth zone, and placental leptin not only contributes to maternal and foetal physiology, but it also regulates placental development, structure, and functions. Related to this, the presence of leptin receptors within the placental tissue, especially located in the syncytiotrophoblast cells as well, indicates that placental leptin exerts autocrine and paracrine functions [8,9,10,11].

Prenatal leptin, as mentioned above, is an important endocrine factor regulating different developmental processes of the foetus itself. Foetal circulating leptin has been proposed to mediate the development and function of several foetal tissues such as the hypothalamus, lung, kidney, and bone, among others, as they have been shown to express leptin receptor in rodent models [4]. To date, it is known that foetal circulating leptin has diverse sources, including maternal circulation, placenta and, in the case of humans, also the foetal adipose tissue [12,13,14,15], although the actual grade of contribution of the aforementioned sources to the levels of foetal circulating leptin is still under debate. In addition to that in foetal circulation, leptin has also been found in other foetal compartments. In this regard, Oliver et al. showed that leptin was present in rat immature stomach during late gestation, specifically in the superficial epithelium of the stomach mucosa [16]. Remarkably, the authors observed a sudden increase of leptin levels in the stomach at delivery, when compared to those of gestational day 19. These high levels did not coincide with the endogenous leptin mRNA expression level, which remained low from day 19 of gestation until 8 h after delivery. Such discrepancy between leptin protein and endogenous mRNA levels drove Oliver et al. to suggest that leptin found in the stomach of late foetuses might be incorporated from other sources rather than being from endogenous production, which was supported by the observation, from immunohistochemistry analysis, that prenatal leptin was located in the superficial epithelium of the stomach mucosa instead of in the basal part of the epithelium [16]. An important remaining question is where this prenatal leptin comes from.

It has been reported that the amniotic fluid (AF) is a source of leptin [17,18], albeit little information exists in the literature on the origin and the physiological role of the amniotic fluid leptin (AFL). AF is produced in the cavity of the amniotic sac, an extraembryonic tissue constituted by the amnion. It acts as a reservoir of nutrients, and it is a dynamic fluid that undergoes constant changes both in the volume and content, in part as a result of the well-established capacity of foetuses to swallow AF, from shortly after week 8 of gestation in the case of the human foetus [19]. It is worth mentioning that, in humans, AF has been proposed to supply leptin to the foetal stomach from the moment that the foetus begins swallowing, and this incorporated leptin may play a role in the growth and functional development of the foetal gastrointestinal tract, probably acting via the long form of the leptin receptor (ObRb), as a strong expression of the ObRb receptor has been observed in the foetal digestive tract during this developmental period [20].

It is plausible to hypothesise that leptin produced by the placenta could contribute to the AFL pool, although there could be other sources, such as amnion cells [21], and that AFL might contribute to a great extent to the levels of prenatal stomach leptin reported in previous studies in rats during the last part of gestation [16]. This seems to be of interest given the role described for leptin present in the stomach at different periods of development. Notably, our research group has previously reported that leptin endogenously produced in the stomach may play a role in the acute regulation of food intake in both humans and rats in adulthood, acting as a satiety signal [22,23,24]. However, in neonate rats and during the suckling period, when the endogenous synthesis of leptin by the immature stomach is still low, the main source of leptin has been shown to be exogenous, from maternal milk, and this leptin may also exert its physiological role in the short-term control of feeding, after its absorption by the stomach and transfer to the general circulation [16,25]. However, the role of leptin ingested during the suckling period may go beyond that of feeding control since it has been shown to be important in the programming of future metabolic health [26]. Indeed, leptin intake at physiological doses during the suckling period in rats has been reported to exert long-term protective effects against the development of obesity and related metabolic alterations in adulthood [27,28]. Indirect evidence from human epidemiological studies also supports the role of leptin, as a breast milk component, protecting against excessive body weight gain in developing infants [26,29]. Thus, the leptin found in the stomach may play an important role in critical windows of development, although its concrete role during foetal life has not been clearly established and deserves more research.

Overall, these findings led us to consider of interest the study of leptin distribution in rat extraembryonic and foetal tissues and fluids at late gestation in order to assess the primary origin of stomach leptin, as well as potential changes in its levels in the different compartments in the days before birth, since this in turn could give us some clues about its possible role. Hence, we specifically aimed (during late gestation, in rats): (1) to study the ontogeny of leptin in the placenta, (2) to gain insights into the potential contribution of placental leptin to the levels of leptin in AF, and (3) to study the possible relationship between AFL and leptin found in the stomach. The resulting findings may bring new insights into the origin of foetal stomach leptin during the perinatal period, which would be important to establish potential strategies to promote appropriate stomach leptin levels for health-promoting metabolic programming. As an experimental model, we studied different extraembryonic and foetal tissues and fluids from rat foetuses in the last four days of gestation (days 18 to 21).

## 2. Materials and Methods

### 2.1. Animal Experimental Design

The animal protocol followed in this study was reviewed and approved by the Bioethical Committee of the University of the Balearic Islands (Resolution number CEEA (*Comité de Ética de Experimentación Animal*, Animal Experimentation Ethics Committee) 43/07/15), and guidelines for the use and care of laboratory animals of the university were followed.

In this study, eight 3-month-old virgin female Wistar rats were mated with male rats. Conception day was confirmed by the detection of sperm in the vagina smear, and it was designated as day 0 of gestation. Pregnant rats were caged individually and housed under standard conditions, including controlled temperature (22 °C), 12 h of light and dark cycle (light on from 8 a.m. to 8 p.m.), and free access to standard chow diet and water. Pregnant rats were distributed randomly into different groups that defined the day of sacrifice. Specifically, 2 pregnant rats were included in each day of sacrifice, from gestational day 18 to 21.

Asphyxia by CO_2_ was the method of sacrifice. Briefly, pregnant rats were introduced into a CO_2_ chamber until confirmation of death by faded eye colour and absence of movement and respiration. Maternal blood was taken afterwards by cardiac puncture to obtain serum. Foetuses were killed by decapitation. Amniotic fluid (AF), umbilical cord (UC), placenta, foetal stomach, and gastric content (GC, liquid inside the stomach) of each foetus were collected, frozen immediately in liquid nitrogen and stored at −80 °C until analysis. Regarding the gastric content, it was collected by sucking the fluid inside the stomach with the BD Micro-Fine syringe coupled to a 30 G needle (Becton, Dickson and Company, Franklin Lakes, NJ, USA) due to the small size of the stomach. Maternal blood samples were centrifuged at 3000 rpm for 10 min at 4 °C, and the resulted serum samples were stored at −80 °C until analysis.

### 2.2. Quantification of Leptin

Stomach, umbilical cord and placenta were homogenised with a disperser homogeniser under cold conditions in 1:3 (*w*/*v*) of phosphate-buffered saline (PBS). For placenta homogenisation, pieces of 100–150 mg from a transversal cut were used, ensuring that the resulting piece contained all the placental layers. In the case of stomach and umbilical cord, pools of 3–5 samples were needed for the homogenisation for technical reasons. Samples for pooling were the same for both stomach and umbilical cord. The homogenate was centrifuged for 5 min at 500× *g* and at 4 °C, and the supernatant was collected. The homogenate was directly used for leptin quantification. Leptin was measured with a mouse/rat leptin enzyme-linked immunosorbent assay (ELISA) kit (R & D Systems, Minneapolis, MN, USA), according to the manufacturer’s instructions. Maternal serum and AF were 1/5 and 1/2 diluted, respectively, prior to leptin measurement. Gastric content samples were also pooled in order to meet the volume requirement of the leptin ELISA kit. Undiluted pools of gastric content were used for leptin determination.

### 2.3. Immunohistochemical Analysis

Immunohistochemistry was performed on five micrometre thick paraffin-embedded transverse sections of placenta at all gestational days (18–21). After dewaxing and rehydration, sections were subjected to a microwaving pre-treatment with citrate buffer (pH 6) for 20 min and then incubated with 5% hydrogen peroxide in water for 10 min to block endogenous peroxidase. Sections were next incubated with 2% normal goat serum in PBS (Vector Laboratories, Burlingame, CA, USA), to block unspecific background, and then with anti-leptin antibody (Abcam, Cambridge, UK, Cat#ac16227), diluted 1:1500, overnight at 4 °C. After a thorough rinse in PBS, sections were incubated in a 1:200 (*v*/*v*) biotinylated secondary antibody solution (in PBS for 30 min). The biotinylated horse-radish peroxidase (HRP)-conjugated secondary antibody (Vector Laboratories, Burlingame, CA, USA) was goat anti-rabbit immunoglobulin G. Finally, sections were incubated with avidin–biotin (ABC) complex (Vectastain ABC kit, Vector Laboratories, Burlingame, CA, USA). Peroxidase activity was revealed by Sigma Fast 3,3-diaminobenzidine as substrate (Sigma Aldrich, St. Louis, MO, USA). Sections were counterstained with haematoxylin and mounted in Eukitt (Kindler, Germany). Images were acquired with Zeiss Axioskop 2 microscope equipped with AxioCam ICc3 digital camera and AxioVision 40V 4.6.3.0. software (Carl Zeiss, S.A., Barcelona, Spain). Staining was never observed when the primary antibody was omitted.

### 2.4. Statistical Analysis

All data are expressed as mean ± S.E.M. Student’s t-test was used for single comparisons between groups when analysing AF samples. Mann–Whitney U test was used for single comparisons when analysing the gastric content, stomach, and umbilical cord. One-way analysis of variance (ANOVA) was used to assess differences among groups (GD18, GD19, GD20, and GD21), followed by least significance difference (LSD) post hoc test. The linear dependence between two variables was assessed using the Pearson correlation test. The threshold of significance was set at *p* < 0.05. Leptin data of the GC, stomach, and UC were weighted prior to statistical analysis by the number of samples pooled during the experimental analysis. The statistical test and the representative symbols used for each comparison are detailed in the footnotes of the tables and figures. The analyses were performed with IBM SPSS Statistics for Windows, Version 23.0. Armonk, NY, USA, IMB Corp.

## 3. Results

### 3.1. Weight-Related Parameters

Dams showed an expected body weight gain throughout pregnancy, and foetuses exhibited a graded increase in body weight as pregnancy advanced (Table 1), indicating that all dams underwent an optimal pregnancy evolution. Placental weight also increased with the gestational days, although the increase was not continued in placentas of 21 days of gestation.

### 3.2. Leptin Levels during Late Gestation in Amniotic Fluid, Gastric Content, Stomach, Placenta, Umbilical Cord, and Maternal Serum

**Figure 1:**
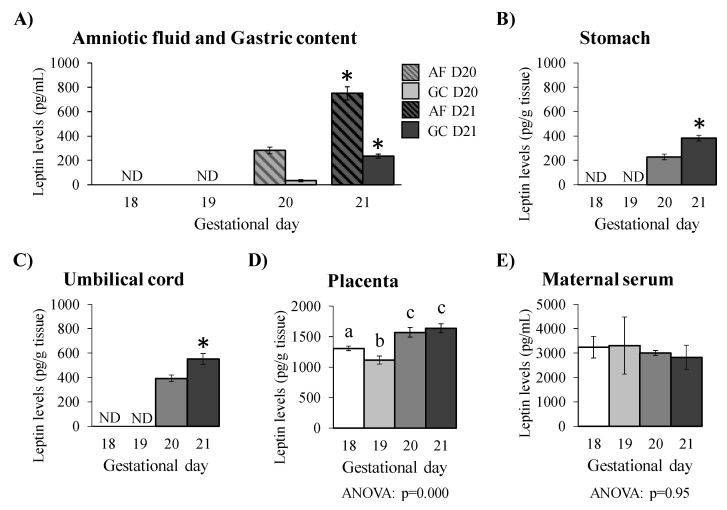
Leptin levels in rat amniotic fluid (AF) and gastric content (GC) (**A**), stomach (**B**), umbilical cord (**C**), placenta (**D**), and maternal serum (**E**) at days 18, 19, 20, and 21 of gestation. Leptin levels in AF and GC have been represented together for a clearer comparison. Pools of stomach, umbilical cord, and GC samples (*n* = 2–5 foetuses per pool) were required for leptin measurement. Data are mean ± S.E.M. (*n* = 22–27 foetuses per gestational day; *n* = 2 dams per gestational day). Statistics: Student’s *t*-test for single comparisons in AF: *, *p* < 0.001 vs. day 20; Mann–Whitney U test was used for single comparisons in GC, stomach, and umbilical cord: *, *p* < 0.001 vs. day 20; LSD post hoc one-way ANOVA test to study the differences between groups: data not sharing a common letter are significantly different, *p* < 0.05. The individual *p*-values from one-way ANOVA are indicated. Abbreviations: D, day; ND, non-detected.

No presence of leptin was detected at 18 and 19 days of gestation in the AF, GC, and stomach of foetuses and in the umbilical cord. However, a sudden appearance of leptin was observed in all the aforementioned fluids and tissues at gestational day 20, followed by a significant increase in its levels at day 21 (Figure 1A–C). Moreover, the Pearson correlation test confirmed the positive and significant linear relationship between leptin levels in all the aforementioned fluids and tissues (Table 2). Interestingly, although it is expected that the GC must primarily be swallowed AF, leptin concentration is considerably lower (by 3–8-fold) in GC than in AF (Figure 1A). In the placenta (Figure 1D), leptin was present at all the studied days of gestation but showed higher levels at days 20 and 21. Leptin levels in the placenta were not correlated with those found in the AF, GC, stomach, and umbilical cord (data not shown). Regarding the maternal serum (Figure 1E), leptin showed similar levels throughout all the gestational days, although we have to point out the small sample size (*n* = 2 dams per gestational day) for maternal serum leptin analysis, which was an accepted limitation of the study, as the objectives were focused on the analysis of foetal and extraembryonic fluids and tissues, which had a size of 22–27 samples per gestational day.

### 3.3. Immunohistochemical Analysis of Leptin in Placenta

**Figure 2:**
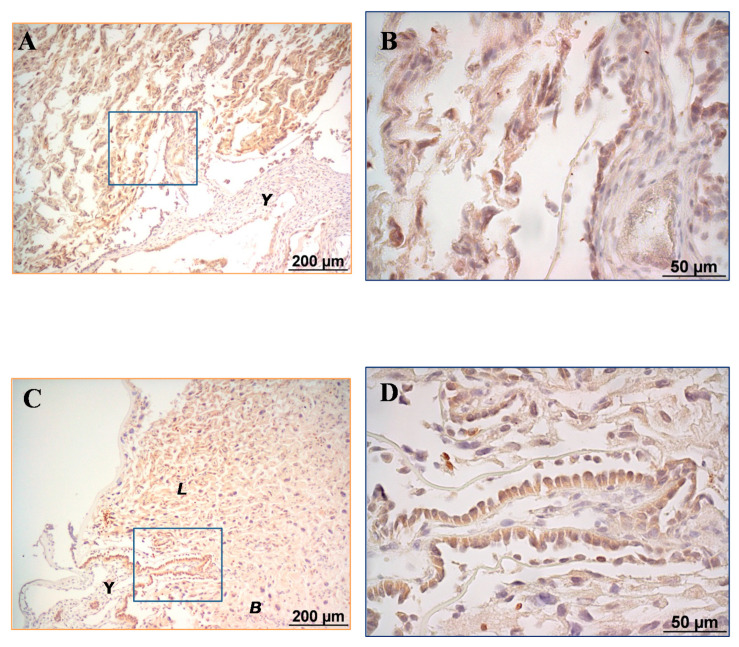

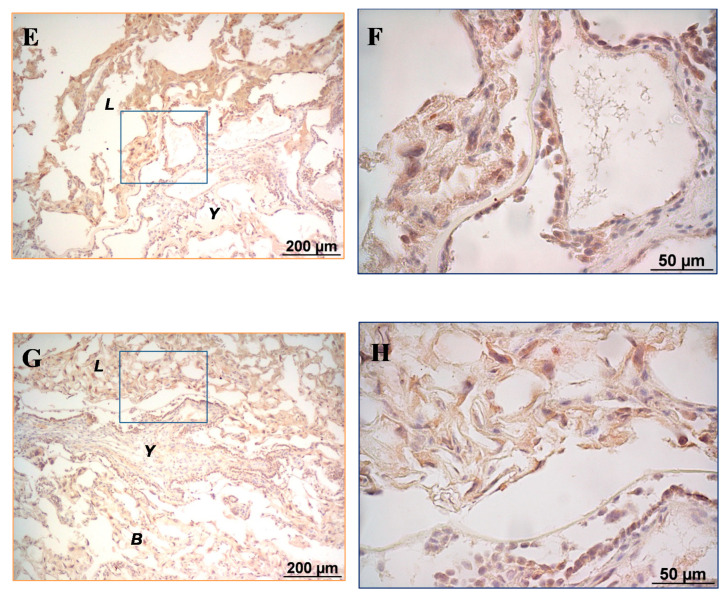
Immunostaining for leptin in the placenta. The images shown are representative of the different groups; the number of image samples analysed in the study was 4, 3, 2, and 2 for days 18, 19, 20, and 21, respectively. The corresponding enlargement of the framed area in (**A**,**C**,**E**,**G**) is on the right (i.e., (**B**,**D**,**F**,**H**), respectively). (**A**,**B**) Placenta of 18 days of gestation. Widespread positivity throughout the placenta and lack of clear separation of the basal and labyrinth zones. Positivity for leptin in the endothelial cells of the visceral yolk sac. (**C**,**D**) Placenta of 19 days of gestation. Visible separation of the basal and labyrinth zones. Placental leptin immunoreactivity is mainly observed in the labyrinth zone. The basal zone is negative for leptin immunoreactivity. Positivity for leptin immunoreactivity in the endothelial cells of the visceral yolk sac remains. (**E**,**F**) Placenta of 20 days of gestation. Same description as in C and D. (**G**,**H**) Placenta of 21 days of gestation. Same description as in (**C**–**F**). Light microscopy, immunohistochemistry of 5 µm thick paraffin-embedded transverse sections of placenta with a rabbit polyclonal anti-leptin antibody (ABC method, primary antibody diluted 1:1500). Scale bar: 200 µm in (**A**,**C**,**E**,**G**); 50 µm in the enlarged area (**B**,**D**,**F**,**H**). Abbreviations: B, basal zone; L, labyrinth zone; Y, visceral yolk sac.

Leptin positivity was localised in the cytoplasm of endodermal epithelial cells of the visceral yolk sac (VYS), the outer foetal origin membrane of the rat placenta that surrounds the foetus [30], at all ages considered (Figure 2).

Leptin was also immunolocalised in the placenta, but the positivity pattern was different among the gestational days. Specifically, leptin positivity was widespread throughout the placenta at day 18 of gestation (Figure 2A,B), which exhibited poor separation of basal and labyrinth layers. However, from gestational day 19, a clear separation of the basal layer and labyrinth was observed (Figure 2C–H), at this moment the immunoreactivity for leptin was evident only in the cytoplasm of the labyrinth cells. The visible distinction of the basal and labyrinth zones at day 19 might be a signal of maturation of the labyrinth zone, which is in accordance with other studies that have evidenced that the labyrinth zone in rat develops with advancing pregnancy [31]. Changes in leptin positivity, from widespread throughout the placenta at day 18 to localised presence in the labyrinth zone from day 19 onwards, might be key in the sudden appearance of leptin at gestational day 20 in the AF, GC, stomach, and umbilical cord.

## 4. Discussion

The participation of adipocyte-derived leptin in the regulation of feeding behaviour and energy balance through the central nervous system in adult mammals is well known and characterised [32,33]. Leptin present in breast milk has also been proposed to play a pivotal role in infants in the programming of future body weight and metabolic health [26], but foetal/perinatal leptin still needs to be investigated in order to get a better understanding of the importance of its physiological role during the perinatal period and the mechanisms involved. To the best of our knowledge, this is the first study jointly analysing the evolution of the levels of leptin throughout the last days of gestation in different extraembryonic and foetal fluids and tissues, as well as the immunolocalisation of leptin in the rat placenta, which could give some clues about its relevance and possible physiological role in such a perinatal stage.

Here we have seen an absence of leptin in AF, gastric content, stomach, and umbilical cord at days 18 and 19 of gestation, followed by a sudden appearance at day 20 and a further increase in its levels at day 21, suggesting that leptin might exert a specific certain role in the regulation of late foetal development (near delivery) or the preparation for birth. Interestingly, our results regarding AFL are similar to the results of other authors showing that leptin was first detectable in rat AF at gestational day 19 and increased greatly at day 21 [34]. We assume that the little discrepancy (day 19 vs. day 20) concerning the detection day of leptin might be a result of the intrinsic range of variability (around 12 h) of the technique used to establish the conception day. The detection of sperm used in both studies is useful to confirm the copulation, but it is not accurate enough to determine the exact time of conception, because the sperm can be detected even several hours after copulation. In this sense, gestational days can actually present a certain margin of difference and, in turn, show differences in the developmental features of the foetuses. However, our results in rats differ from those observed in humans. More specifically, human leptin levels in AF have been reported to be significantly higher at mid-gestation than at late gestation [18,20]. These discrepancies might also reflect intrinsic differences between rodents and humans regarding leptin levels in AF. This can be relevant as, for instance, the ontogeny of adipose tissue development is different between rodents and humans, taking place earlier in humans (mainly during late gestation) than in rodents (especially during the first postnatal weeks) [35,36]. Coming back to AFL, despite these interspecies differences regarding AFL level progression, it is interesting to point out that leptin present in the AF might be important for correct foetal growth and development in both species, and we also speculate that the rise in AFL by mid-gestation in humans and by late gestation in rats might have a specific role. Interestingly, in humans, it has been suggested that high levels of leptin in AF during the second trimester may be involved in the developmental process of the digestive tract when it gets in contact with the digestive mucosae, after the swallowing of the AF, and probably through the leptin–ObRb complex [20].

As described by Oliver et al., leptin found in the rat foetal stomach could have an origin from other foetal or extraembryonic compartments, such as AF, rather than being from endogenous production since leptin mRNA expression pattern differs from that of protein levels at late gestation [16]. In our study, it seems quite logical that the GC collected must be swallowed AF, and that the source of leptin present in the GC may be from the swallowed AF, as reflected in the leptin levels measured in the GC samples of gestational days 20 and 21. As mentioned, GC and AF display a similar pattern in leptin levels in the last two days of gestation (days 20 and 21), but leptin levels in GC are considerably lower than those in AF (by 3–8-fold). This decrease might suggest a “leptin loss” from GC, probably by its internalisation to the stomach mucosa. Moreover, the fact that leptin levels in all AF, GC, and stomach are positively correlated, would support the hypothesis of AFL internalisation/absorption by the immature stomach of near-term rat foetuses. Interestingly, absorption of orally given leptin by the immature stomach mucosa, either from maternal milk or given as a supplement, has already been reported in neonate rats [16,25]. Given the role in short-term feeding regulation that has been attributed to gastric leptin, also including the leptin up taken by the immature stomach during the suckling period [23,25,37], the possible implications of AFL internalised into the gastric mucosa deserve further research, particularly when considering the sudden appearance of leptin (and following level increase) at day 20 of gestation reported here.

Among the possible main physiological targets of the internalised leptin from the gastric content, apart from the gastrointestinal tract itself as suggested above (through the leptin–ObRb complex in the digestive mucosae [20]), the hypothalamus could also stand out as a possible candidate. We have to consider that, on the one hand, the hypothalamus is key in the central regulation of feeding behaviour and energy expenditure throughout life [38]. On the other hand, leptin has been shown to play a crucial neurotrophic role in hypothalamic development, particularly in rodents during the first two weeks of life; moreover, the influence of leptin in the development of the human hypothalamus, which occurs during pregnancy, has also been suggested [39,40,41]. Supporting this, our research group has previously shown that oral leptin supplementation, at physiological doses during lactation, prevents obesity in adult rats, even under a high-fat diet, in part as a result of particular changes in the structure and function of the hypothalamus, giving out a more protective phenotype against later obesity development [27]. Moreover, leptin supplementation during the suckling period has been shown to ameliorate alterations in hypothalamic structure and function due to adverse gestational conditions [42], as well as alterations in sympathetic drive in the adipose tissue [43] and the stomach [44]. However, whether AFL internalised by the stomach may be relevant for the development of these key structures and tissues requires more (specific) research. It is worth mentioning that, to date, the neurotrophic actions of leptin during the perinatal period have been primarily associated with circulating leptin [45,46]. Therefore, the study of the possible contribution of non-circulating leptin in developmental processes may provide new insights into the important trophic role of this hormone. Moreover, aside from its potential role in the development, it could be hypothesised that the sudden increase in AFL levels found at late gestation may be important in the transition from in utero to postnatal life. The intake of a certain amount of leptin from the AF and, consequently, the presence of greater amounts in the stomach, could provide a signal of satiety around birth, as previously suggested [17], while the subsequent decrease in stomach leptin levels shown shortly after birth, during the first postnatal day [16], may be interpreted as a stimulus for the starting of sucking [47]. The observation in previous studies of a lack of stimulation of endogenous leptin production in the stomach of neonate rats from 4 to 8 h of life in pups that were separated from their mothers immediately after birth and deprived of suckling [16], supports the role of gastric leptin in feeding regulation during the perinatal period. Overall, the present data lead us to hypothesise that prenatal leptin found in the stomach might exert physiological functions in the early control of food intake and possibly also in the hypothalamus development during the last part of gestation, which is key in the central regulation of feeding [46].

After the presence of leptin in AF and GC was confirmed and the suggestion about the possible origin of the rat foetal stomach leptin (as leptin internalised from the swallowed AF), new questions regarding the source of AFL rose up. It is well known that maternal serum leptin levels are elevated during gestation, leading to the idea that maternal leptin could be a source of AFL. In humans, the correlation between maternal circulating leptin and AFL is controversially demonstrated, as there are studies showing both the presence and absence of correlation. More specifically, in different studies, a correlation of maternal serum leptin with AFL has been found in general [17], or only in neural tube defect pregnancies [48], or even a lack of correlation [49] has been reported. The lack of consistency in the results from the different studies could be due to several factors, such as the experimental design itself, the sample size, ethnic differences, etc. Therefore, a standardised protocol for the study of prenatal leptin might be required in order to minimise inter-study variability. In our animal study, the correlation test was not performed because of the large difference in the sample size between maternal serum and AF. However, taking into account that leptin was detectable in maternal serum at all the gestational days studied, displaying similar levels throughout the studied days, unlike what was found in the AF, it is reasonable to suggest that the sudden appearance of leptin in the AF at day 20 must be primarily related with another specific source, and the placenta might be one of the most relevant candidates, since this is an important source of leptin during gestation [10,50].

Therefore, the assessment of the ontogenic presence of leptin (during late gestation) in the placenta and the investigation of its possible contribution to AFL levels was also an objective of the present study. For the first time, the presence of leptin in rat placenta as well as its variation throughout the last part of gestation has been shown by immunohistochemistry. More specifically, we showed that at day 18 of gestation the immunoreactivity for leptin is diffuse and widespread throughout the placenta. In contrast, from gestational day 19 onwards, the presence of leptin predominates in the labyrinth zone. Interestingly, the restricted presence of leptin to the labyrinth zone is shortly prior to the sudden appearance of leptin in the AF (day 20 of gestation), leading us to speculate a possible secretion of placental leptin to the AF in this developmental period, by an as yet unknown mechanism that deserves to be studied. Nevertheless, it is interesting to point out that the expression of the leptin receptor isoform a (ObRa), involved in the transport of leptin through physiological barriers, in the labyrinth zone has been reported to significantly increase in the last part of gestation. In contrast, this increase is not produced in the basal zone [12]. In this sense, the transport of placental leptin to other compartments mediated by ObRa could be suggested as a potential mechanism. It is worth mentioning that the presence of leptin in the placenta, observed by immunohistochemistry, at all studied gestational days, is in accordance with results from placental leptin quantification, which also show the presence of leptin from day 18 to day 21 of gestation. The appearance of leptin in the AF at a precise gestational moment (day 20 of gestation) might be related with certain short- and/or long-term physiological functions. In this regard, considering that the transfer of leptin from the stomach to the bloodstream has been previously described in neonate rats [25], the possible internalisation of AFL by the stomach of near-term foetuses, and its putative posterior transfer to the foetal circulation, should be worth being considered when investigating the functional role of AFL during late gestation, as well as its potential impact on future health. It should be pointed out that results from immunohistochemistry only propose a certain association between placental and AF leptin, but they do not robustly demonstrate whether the placenta actually contributes to the AFL. Hence, further studies are required to explore the actual contribution of the placenta to AFL, the possible mechanisms involved, and their regulation.

In this study, immunoreactivity for leptin has also been observed for the first time in the cytoplasm of endothelial cells of the visceral yolk sac (VYS). The fact that leptin positivity was found in the VYS at all ages considered (from day 18 to 21 of gestation), with no apparent immunostaining changes among the different days, suggests that VYS leptin is independent of the grade of placental maturation at late gestation, and might not be directly dependent on placental leptin production. To date, little is known about the presence and role of leptin in the rat VYS. In line with the reported presence of leptin receptor in murine yolk sac cells during the second week of gestation (days 8–14), we suggest that the leptin found in VYS at all considered ages might exert an auto/paracrine role, as the yolk sac is functional until parturition in rodents [30,51,52]. Specifically, VYS contributes to the transport, absorption, and digestion of nutrients until immediately before parturition in rodents, unlike human’s yolk sac that undergoes a degenerative process until leaving no remnant at birth [53,54]. Taking into account the reported presence of leptin receptor in murine yolk sac, the functionality of the yolk sac during the whole gestation and the presence of leptin during late gestation seen in our study, we suggest that leptin might exert a certain role in the regulation of yolk sac functions, which in turn might impact foetal growth.

Regarding the leptin determined in the umbilical cord, we found the limitation of the impossibility to distinguish whether the leptin came from foetal blood, maternal blood, or only from the cells of the cord itself, as a mix of these components was finally used for the measurement of leptin levels due to the technical restrictions at the moment of tissue collection. However, it is interesting that, as in AF, leptin was not detected in the umbilical cord until day 20, and we cannot rule out the possibility that it could provide part of the leptin found in the AF.

## 5. Conclusions

All in all, we conclude that leptin in the AF is first detectable at day 20 of gestation in rats followed by a significant increase at day 21, and its concentration is correlated with the leptin concentration in the foetal stomach. The correlation of AF and stomach leptin, and the several-fold decrease in leptin levels in the GC, when compared to those of the AF, might suggest the internalisation of AFL to the immature stomach after AF swallowing. Future studies would be needed to clarify the hypothetical destination and impact of the internalised stomach leptin on the offspring’s metabolic health, as perinatal leptin is key to short-term control of feeding and metabolic programming. This is the first study showing the presence of leptin in the placenta and VYS of rats by immunohistochemistry, as well as changes in the location of leptin immunoreactivity in the placenta during the last part of gestation. More specifically, we show the transition from a diffuse presence of leptin in the placenta, seen at gestational day 18, to a well-defined leptin localisation in the labyrinth zone from day 19 until immediately before parturition. Finally, these changes in the placenta might help to explain the sudden appearance of leptin in the AF at day 20 of gestation, although the mechanisms involved are unknown and deserve further investigation.

## Figures and Tables

**Table 1:** Weight-related parameters. (**A**) Dams’ body weight at sacrifice (gestational day (GD) 18 to 21) and body weight gain (calculated as the increase of body weight from conception day (day 0) to final weight at day of sacrifice). (**B**) Body weight, placenta weight, and number of foetuses at sacrifice (GD 18 to 21).

			Gestational Day			
			18	19	20	21
(A)	Dams	Body weight (g)	323.5 ± 1.6	321.4 ± 0.1	338.7 ± 2.1	345.3 ± 1.4
		Body weight gain (g)	102.4 ± 3.4	87.7 ± 0.4	99.1 ± 3.2	102.7 ± 1.4
(B)	Foetuses	Body weight (g)	1.23 ± 0.02 a	2.11 ± 0.04 b	3.28 ± 0.06 c	4.843 ± 0.11 d
		Placenta weight (g)	0.30 ± 0.01 a	0.42 ± 0.01 b	0.48 ± 0.01 c	0.44 ± 0.01 b
		Total number of foetuses (foetuses per dam)	27 (11; 16)	23 (10; 13)	23 (9; 14)	24 (12; 12)

Data are mean ± S.E.M. (*n* = 2 per GD in the case of dams, and *n* = 23−27 per GD in the case of foetuses). Statistics: least significant difference (LSD) post hoc one-way ANOVA test to study the differences between foetal groups: data not sharing a common letter are significantly different, *p* < 0.05.

**Table 2:** Correlation matrix of leptin content in amniotic fluid, gastric content, stomach, and umbilical cord.

Sample	Amniotic Fluid	Gastric Content	Stomach	Umbilical Cord
Amniotic fluid	1			
Gastric content	0.688 **	1		
Stomach	0.598 **	0.715 **	1	
Umbilical cord	0.603 **	0.537 **	0.515 **	1

The Pearson’s correlation coefficient is given. Correlations were performed with samples from gestational days 20 and 21. Samples of each fluid/tissue were pooled for leptin measurement, except amniotic fluid samples. In the case of stomach and umbilical cord (*n* = 6–10 pools), 2–3 samples were pooled, while in the case of gastric content (*n* = 2–3 pools), pools of 3–5 samples were required for the determination. ** Significant correlation at *p* < 0.01.
